# Short versus long-acting erythropoiesis-stimulating agents for anemia management in Egyptian hemodialysis patients

**DOI:** 10.5339/qmj.2024.16

**Published:** 2024-03-11

**Authors:** Amira Elsawy Soliman, Salma Magdy, Hazem Sayed Ayoub, Abdel-Hameed Ibrahim Ebid

**Affiliations:** 1 Faculty of Pharmacy, Helwan University , Helwan, Egypt Email: salma.magdy@pharm.helwan.edu.eg; 2 Faculty of Medicine, Al-Azhar University, Cairo, Egypt

**Keywords:** Short-/long-acting ESAs, hemodialysis, anemia, chronic renal failure, pharmacoeconomics

## Abstract

Background: Chronic kidney disease (CKD) often results in renal anemia, impacting the well-being of patients and causing various negative consequences. Erythropoiesis-stimulating agents (ESAs) offer promising solutions for managing anemia in CKD. This study aimed to evaluate and compare the effectiveness, safety profile, and cost-effectiveness of short-acting (Eprex^®^) and long-acting (Aranesp^®^) ESAs.

Method: This comparative prospective cohort cost-effectiveness study was carried out over 6 months among adult Egyptian hemodialysis patients of either gender. Participants were categorized into two groups based on the type of ESA administered: the Eprex group, receiving epoetin alfa, and the Aranesp group, receiving darbepoetin alfa. These two treatment groups’ efficacy, safety, and cost were analyzed and compared.

Results: Of 127 hemodialysis patients, 60 (47.2%) received Eprex, while 67 (52.8%) were treated with Aranesp. Target hemoglobin (Hb) was achieved by 50.6% of patients in the Eprex group versus 63.4% in the Aranesp group, with a significant difference (*P* < 0.001). Both treatment groups exhibited a similar safety profile, while Aranesp^®^ was considered the cost-saving protocol.

Conclusion: In hemodialysis Egyptian patients, Aranesp with extended dosing intervals proved to be more effective in achieving target Hb with comparable adverse effect profiles, a substantial cost-saving strategy, and offered time-saving advantages for medical staff workload compared to Eprex.

Trial registration: The Clinicaltrial.gov registration ID is NCT05699109 (26/01/2023).

## Introduction

Chronic kidney disease (CKD) stands as a leading cause of global mortality in the 21st century, with significant risk factors, including diabetes mellitus, hypertension, and obesity. Recent updates as of 2022 indicate that CKD has affected over 800 million people worldwide, with a growing prevalence.^1^ In Egypt, the estimated yearly incidence of CKD is 74 cases per million, with a total of 264 cases per million of the population (pmp) undergoing dialysis.^2^

Anemia represents an inevitable complication of CKD caused primarily by the inability of the failing kidney to produce adequate erythropoietin to stimulate the production of red blood cells (RBCs).^3^ Anemia of CKD is linked with increased mortality, cardiovascular disease, fatigue, dyspnea, and diminished patient quality of life.^4–6^ Erythropoiesis-stimulating agents (ESAs) have revolutionized the management of anemia of CKD by elevating hemoglobin (Hb) levels and improving patients’ quality of life.^7,8^ In Egyptian hemodialysis centers, the most commonly utilized ESAs are Epoetin alfa (Eprex^®^), a shorter half-life administered typically one to three times weekly, and Darbepoetin alfa (Aranesp^®^), known for its longer half-life and reduced dosing frequency (once a week or biweekly).^9–11^ Extending the dosing interval of Aranesp^®^ introduces benefits to patients and healthcare providers, such as time-saving, compared to Eprex^®^.^12^

Till now, it has remained a point of divergence among previous clinical studies^12–20^ regarding whether Aranesp^®^ and Eprex^®^ exhibit similar efficacy and safety profiles or if one attempts to outdo the other. However, it is crucial to note the significant price disparity between the two: a single prefilled Eprex^®^ 4000 IU/ml unit costs 332.5 LE, whereas a single prefilled Aranesp^®^ 40 mcg syringe is priced at 902.5 LE.^21^ Therefore, this study aimed to compare the efficacy, safety, and cost-effectiveness of Eprex^®^ and Aranesp^®^ to help us decide which should be preferred for use in Egyptian hemodialysis centers.

## Patients and Method

### Study Population

A comparative prospective cohort cost-effectiveness study was conducted on hemodialysis patients with CKD at the dialysis units at the Memorial Souad Kafafi University Hospital, Giza, Egypt, between January 1, 2021, and September 31, 2021.

The enrolled patients were diagnosed with CKD in a time range of 3–5 years and started hemodialysis within only 1 year and utilized Fresenius dialysis machines with bicarbonate as a dialysate. Patients were eligible for this study if they fulfilled all the following inclusion criteria: (1) stable receiving regular hemodialysis three times per week for a minimum of 3 months, (2) 18 years or older, and (3) receiving a single type of ESA treatment, either short-acting epoetin alfa (Eprex^®^, Johnson & Johnson, Beerse, Belgium^21^) or long-acting darbepoetin alfa (Aranesp^®^ Amgen, California, United States^22^) for a minimum of 3 months prior to the start of the study.

Eprex, as recombinant human erythropoietin (rHu-EPO), is a glycosylated protein hormone with a molecular weight of 30,400 daltons. The molecule contains 40% carbohydrates and 60% amino acids by mass. In contrast, Aranesp has a molecular weight of 37,100 daltons, a 51% carbohydrate makeup, and 5 N-linked oligosaccharide chains. Additional carbohydrates contribute to its longer half-life and enhanced biological activity.^23^

Patients were excluded from this study if they met any of the following exclusion criteria either within 3 months before the study or during the study period: (1) change in the type of ESA treatment; (2) underwent major surgical surgery or received RBC transfusions; (3) demonstrated non-compliance with dialysis treatment by missing more than two sessions monthly; (4) were infected with COVID-19; (5) had a malignancy diagnosis; (6) underwent kidney transplantation; (7) were pregnant or breastfeeding women; and (8) failed to be followed up for a 6-month study period. Before enrollment, written informed consent was obtained from all patients. Eligible patients were assigned to one of two groups based on their ESA treatment type: Eprex^®^ (4000 IU/ml) or Aranesp^®^ (40 mcg). Subsequently, eligible patients were prospectively followed up for 6 months.

### Study Protocol

The study protocol was approved by the hemodialysis unit at the Memorial Souad Kafafi University Hospital, Giza, Egypt, and the ethical committee at the Faculty of Pharmacy, Helwan University, Cairo, Egypt [approval number: 05H2022 (11/12/2022)]. Routine clinical practices in the unit adhered to standard guidelines as per KIDIGO.^24^ All patients had received ESA treatment either (Eprex^®^) or (Aranesp^®^) subcutaneously for their anemia management. At the beginning of treatment, an initial dose of ESA was calculated based on the patient’s weight, ranging from 100 to 150 IU/kg/week and 0.5 mcg/kg/week for Eprex and Aranesp, respectively. Eperx was administered one to three times weekly, whereas Aranesp was administered once weekly or once every 2 weeks. The dose and frequency of ESA were adjusted monthly to achieve a target Hb level of 10.0–11.5 g/dl. If the patient’s Hb levels fell within the ranges of up to 11 g/dl, 11–11.5 g/dl, and more than 11.5 g/dl, the frequency of Aranesp administration would be once per week, once per 2 weeks, and none per that month, respectively. For Eprex, the corresponding frequencies would be three times per week, twice weekly, and stop after 2 weeks, respectively, for the same Hb levels.

Additionally, all patients received L-carnitine and vitamin B complexes during each dialysis session. Concurrent medications based on patients’ comorbidities were documented, and intravenous iron was prescribed for patients with a transferrin saturation (TSAT) of ≤ 30% and a ferritin level of ≤ 500 ng/ml (≤ 500 mg/l).^24^ IV iron formulations are used as they are superior to oral agents in treating iron deficiency anemia, especially in patients who need hemodialysis. The effectiveness of oral iron agents can be reduced by diminished iron absorption in the gastrointestinal tract and a high rate of gastrointestinal adverse effects.^25^

### Data Collection

Data on various demographic characteristics, including gender and age, as well as body weight, height, the etiology of CKD, vascular access type, concurrent medications, and various comorbidities, were recorded at the start of the study. According to hospital protocol, pre- and post-dialysis body weight, pre- and post-dialysis blood pressure, dialysis duration, and laboratory data were recorded in continually updated follow-up medical records. Dialysis efficacy, quantified using the (Kt/v) parameter, was retrieved directly from the hemodialysis machine.

Periodic, regular laboratory tests, including Hb, creatinine, calcium, phosphate, sodium, and potassium measurements, were performed each month before the hemodialysis session. Additionally, assessments of ferritin, TSAT, parathyroid hormone levels, and albumin were conducted at 3-month intervals. During the monthly visit over 6 months, adverse events like pain at the site of injection, nausea, vomiting, diarrhea, edema, and any other adverse events that might occur were monitored and recorded. Blood pressure measurements were taken both before and after each dialysis session.

### Patients’ Adherence

At the end of the study period, self-reported adherence behavior was assessed using a validated and reliable questionnaire (End-Stage Renal Disease Adherence Questionnaire: ESRD-AQ). In this study, the ESRD-AQ was employed to assess the adherence and counseling practices of the participants toward their HD treatment. All patients were interviewed using the ESRD-AQ, which comprises 46 items categorized into five sections: patient general information, HD session attendance, adherence to medications, fluid restriction, and diet instructions.^26^ The Arabic-translated version of the ESRD-AQ, as provided by Naalweh et al.,^27^ was utilized.

According to ESRD-AQ, higher scores reflect stronger adherence to measured behaviors. Specifically, a total score for adherence behavior falling below 700 was interpreted as poor or non-adherence, while scores ranging from 700 to 999 indicated moderate adherence. Scores within the 1000–1200 range signified good adherence.^27^ More details of ESRD-AQ’s items, subscales, and scoring were described by Kim et al.^26^

### Study Outcomes

The primary efficacy outcome was to measure the changes in Hb level from baseline over the 6-month follow-up in both groups. The secondary efficacy outcome was to assess the percentage of patients in each group who achieved the target Hb (10.0–11.5 g/dl). Safety was evaluated by monitoring the incidence and severity of adverse events reported by patients in both groups during each visit. A cost-effectiveness analysis from a healthcare system perspective was done by calculating the incremental cost-effectiveness ratio (ICER) using the following formula:^28^

$${\text{ICER = Cost}}_{\text{Aranesp}}{\text{ - Cost}}_{\text{Eprex}}{\text{/Effectiveness}}_{\text{Aranesp}}{\text{ - Effectiveness}}_{\text{Eprex}}$$

Cost data, including the expenses associated with ESA, iron therapy costs, hemodialysis sessions, adjuvant therapy, monthly medical follow-up costs, and adverse events, were collected from the financial records of The Memorial Souad Kafafi University Hospital. Costs were collected in Egyptian pounds (LE), and only direct medical costs were included in the analysis. Effectiveness was determined by calculating the mean 6-month Hb measured during patient follow-up.

### Sample Size and Statistical Methods

The sample size for this comparative prospective clinical study was determined using Epi-calc 2000, considering evidence from a similar study conducted by Bernieh et al.^16^ Assuming 80% power and a significance level of 0.05, the initial sample size was calculated to be 90 participants, with 45 in each treatment group. To account for potential dropouts, a 10% dropout rate was considered. Therefore, this study’s final sample size was 100 participants, with 50 individuals in each treatment group.

SPSS version 26 (IBM, Armonk, New York, United States) was utilized for all statistical data analysis. Descriptive statistics, including arithmetic mean and standard deviation, were used to summarize normally distributed data. In contrast, median and interquartile ranges were utilized for summarizing data that did not follow a normal distribution. Qualitative data were presented using frequencies. To assess significant differences among the groups in terms of continuous variables, an unpaired Student’s *t*-test was applied for normally distributed data. At the same time, the Mann–Whitney test was used for non-normally distributed data. A chi-square test was employed for categorical data to compare differences among ESA users. The significance level was set at a two-sided *P*-value of ≤ 0.05.

## Results

### Patient Disposition and Baseline Characteristics

**Figure 1.**
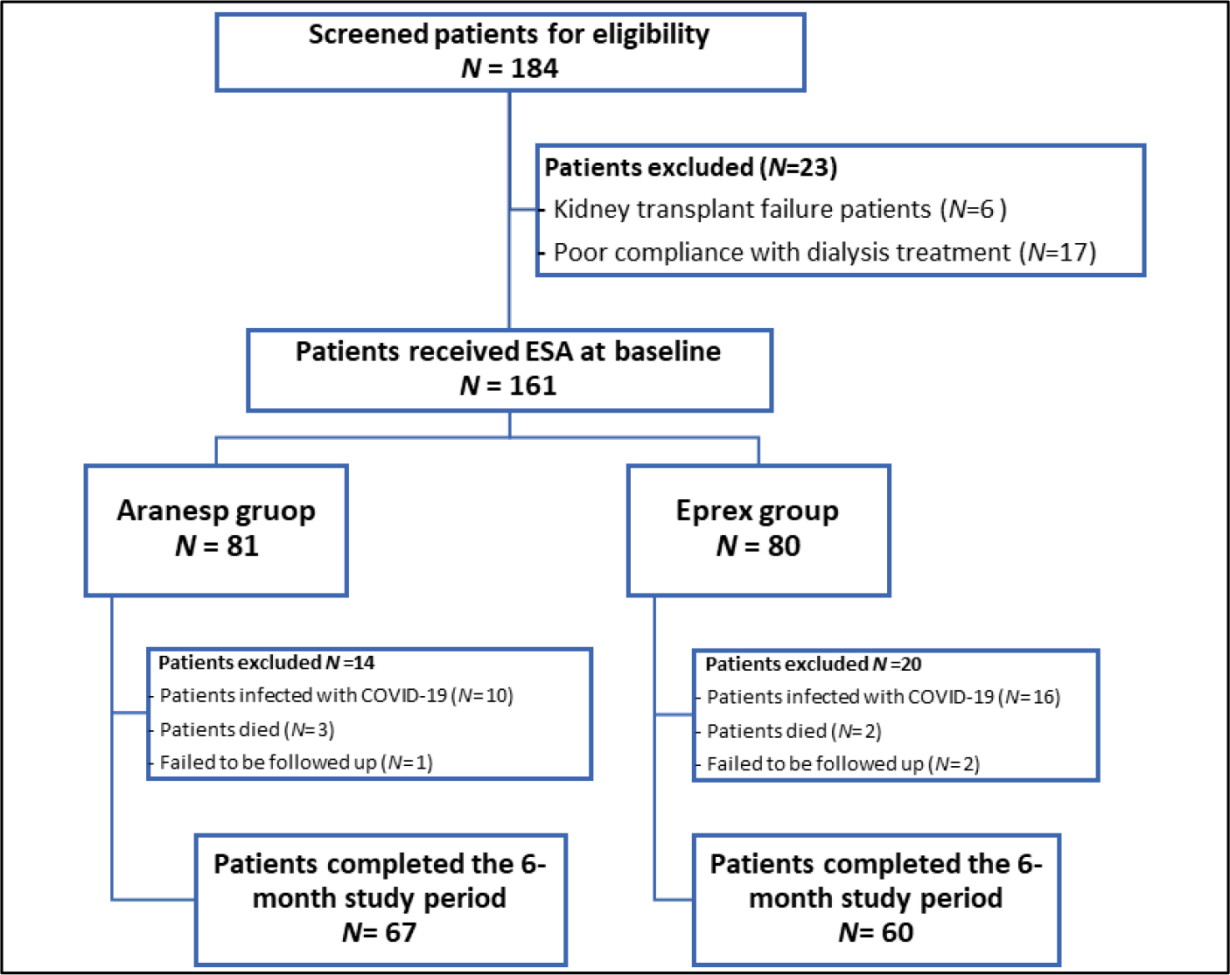
Study flow chart.

A total of 184 patients had been screened, of whom 23 were excluded from the study. Subsequently, 161 who met the eligibility criteria were enrolled and assigned to either the Aranesp (*n* = 81) or Eprex group (*n* = 80). Ultimately, the study analysis included 67 patients from the Aranesp group and 60 from the Eprex group (Figure 1).

At baseline, the demographic characteristics of both ESA treatment groups were similar, with no significant differences observed in gender, age, weight, smoking status, etiology of renal disease, hepatitis status, or vascular access (*P* > 0.05). Most studied patients (*n* = 83, 65.4%) in both groups had a mean age of 60.9 (± 9.5) years. Hypertension was the major leading cause of ESRD in both groups and was encountered in 57 (44.9%) of the total patients. Most patients (*n* = 89, 70.1%) were negative for viral hepatitis, while 38 patients (29.9%) tested positive for HCV. Vascular access was predominantly through an arteriovenous fistula (AVF) in 109 cases (85.8%), with 18 cases (14.2%) utilizing a permacath catheter (Table 1).

No significant difference was found between the two treatment groups in all baseline biological parameters (*P* > 0.05). However, when comparing the mean values of biological parameters during the 6-month study period, only Hb exhibited a significant difference between the two groups (*P* < 0.001) (Table 2).

### Patients’ Adherence

All 127 patients from both treatment groups participated in interviews using the validated and reliable ESRD-AQ questionnaire. The total adherence behavior score indicated good adherence for all patients, as each participant’s total score exceeded 1000. Furthermore, no statistically significant difference was observed between the two treatment groups (*P* > 0.05).

### Efficacy Assessment

#### Primary Efficacy Analysis

**Figure 2.**
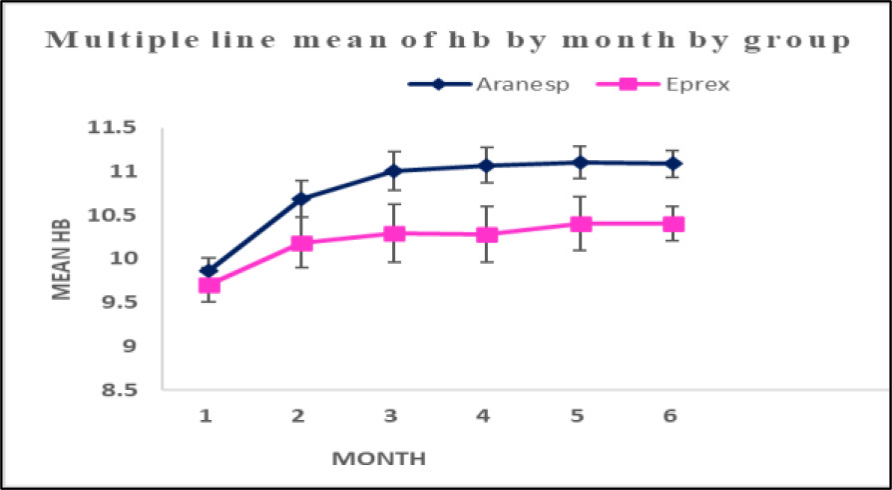
Mean Hb levels change in both treatment groups over the 6-month study period.

**Figure 3.**
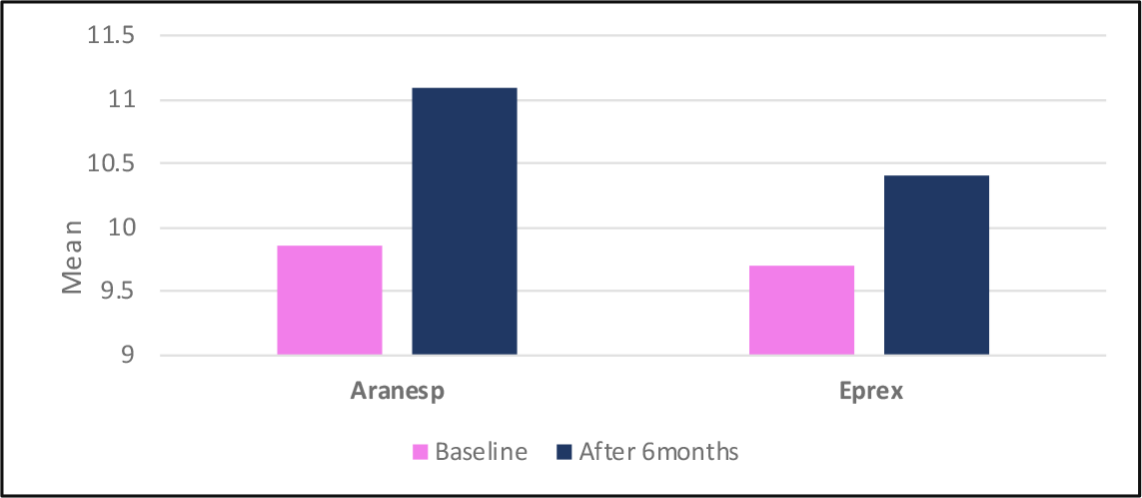
Comparison of baseline and after 6 months Hb levels.

Hb level elevations in both treatment groups were observed during the study period, with a significant difference in both treatment groups individually from baseline to the sixth month (Figure 2). The mean difference in Hb between the first and sixth months was 1.2 and 0.7 g/dl in the Aranesp and Eprex groups, respectively (Figure 3). It was shown that Aranesp had equivalent efficacy to Eprex (*P* > 0.05) at baseline and significantly greater efficacy than Eprex (*P* < 0.001) at the end of the study period (Table 3).

#### Secondary Efficacy Analysis

The target Hb (10–11.5 g/dl) was achieved by 63.4% of patients in the Aranesp group and 50.6% in the Eprex group. There was a significant difference between the two groups in achieving this target Hb range (*P* < 0.001). Detailed percentages of Hb levels within different ranges are provided in Table 4.

### Safety Assessment

During the 6-month study period, a total of 18 patients (26.9%) in the Aranesp group and 25 (41.7%) in the Eprex group reported experiencing at least one adverse event. It is important to note that a single patient could have reported more than one adverse event. Additionally, no hypersensitivity reactions were observed in either group, and there was no statistically significant difference between the two groups in terms of adverse events (*P* > 0.05), as detailed in Table 5.

### Cost Assessment

#### Total ESA Cost

The median total cost for Aranesp over 6 months was 21,660.0 LE (ranging from 12,635.0 to 21,660.0 LE), whereas for Eprex, it was 23,940.0 LE (ranging from 21,280.0 to 23,940.0). The median cost of Aranesp was 2280.0 LE less per 6 months than that for Eprex, and this difference was statistically significant (*P* < 0.001).

#### Cost-Effectiveness Analysis

**Figure 4.**
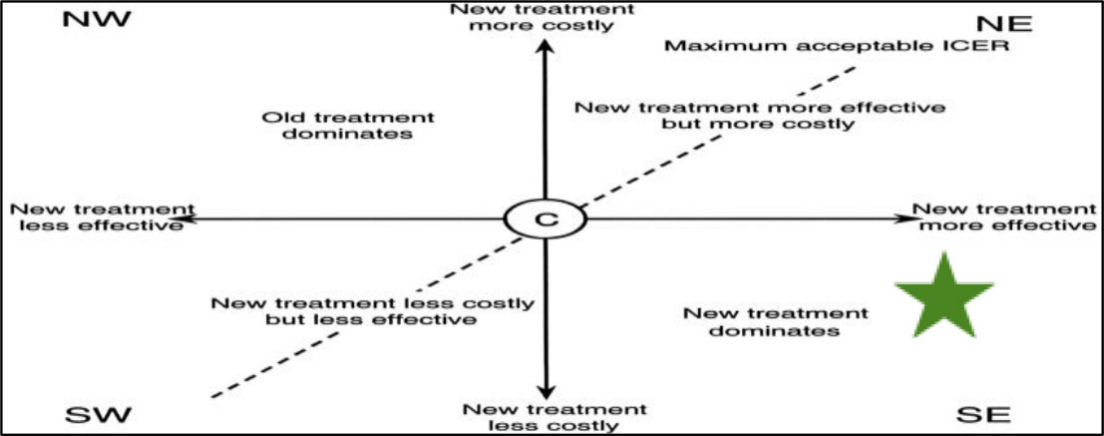
The effectiveness plane.

The total cost of Aranesp, including drug costs, iron costs, hemodialysis sessions, adjuvant therapy, medical monthly follow-up costs, and adverse events, was estimated to be 6,356,045 LE over 6 months, with an average of 94,866 LE per patient per 6 months. The patients averaged the costs due to the unequal number of patients in the two treatment arms. In contrast, the total cost of Eprex, including the same items, was estimated to be 6,110,000 LE over 6 months, with an average of 101,833 LE per patient. The calculated ICER was −13,934 LE per unit of effectiveness, indicating that Aranesp is dominant (a cost-saving strategy) according to the cost-effectiveness analysis. The result of the ICER was plotted against the cost-effectiveness plane (Figure 4).^28^

## Discussion

ESAs have proved effective in managing anemia in millions of CKD patients, significantly reducing blood transfusion requirements.^8^ This study was a comparative prospective cohort cost-effectiveness study aimed to evaluate and compare the efficacy, safety, and cost of short-acting Eprex^®^ versus long-acting Aranesp^®^ in treating renal anemia among Egyptian patients with ESRD undergoing hemodialysis. Both Aranesp^®^ and Eprex^®^ are the most extensively utilized ESAs under governmental health insurance in Egypt to correct anemia in hemodialysis patients.

In this study, all demographic data and biological parameters evaluated throughout the study were comparable between the two treatment groups, and there were no statistically significant differences observed in these parameters between the baseline and end of the study. Moreover, the patients studied were 18 years of age or older to reduce the variability by studying the adult age group and ensuring a reliable conclusion. We also excluded any patient who received a blood transfusion from our study to avoid any conflict in the results. Blood transfusion was suggested in severe recalcitrant conditions that necessitate urgent blood transfusion that would lead to an elevation in Hb level, not due to the studied agents.^29^ This equivalence between the two groups was achieved as they were carefully matched for several critical confounding factors.

We confirmed that all 127 patients from both treatment groups exhibited good adherence using the validated and reliable ESRD-AQ questionnaire.^26^ Patients’ adherence in clinical studies should be confirmed, as non-adherence leads to failure to detect an actual treatment effect and the risk of overestimating drug safety.^30^

The KDIGO Clinical Practice Guideline for Anemia in CKD recommends that CKD patients with anemia receiving ESAs should not aim to maintain a Hb level above 11.5 g/dl in adults. Instead, an acceptable target range for Hb is suggested, typically falling between 10 and 11.5 g/dl.^24^ This range is the optimal target in hemodialysis patients and is recommended to mitigate the potential cardiovascular complications and mortality risks associated with exceeding the upper limit.^24,31^ Over this 6-month study period, both Aranesp and Eprex demonstrated significant increases in Hb levels from baseline. However, with its extended intervals, Aranesp effectively stabilized patients’ Hb levels within the desired target range and exhibited a more substantial correction effect on anemia compared to Eprex, which had shorter dosing intervals. Notably, the Aranesp group showed a significantly greater efficacy with a mean Hb difference of 1.2 g/dl between the first and sixth months, in contrast to the 0.7 g/dl difference observed in the Eprex group (*P* < 0.001).

Moreover, our results emphasized the ESA type’s impact in achieving and maintaining target Hb. There was a significant difference in the percentage of patients achieving target Hb in the Aranesp group compared to the Eprex group. These findings align with previous studies.^16–18^ However, it is worth noting that other published studies have concluded that one ESA formulation has no superiority over another.^12,19,20,32^ These discrepancies may arise from differences in study designs and the multicenter nature of those studies.

From a mathematical perspective, when Eprex is administered three times a week, it requires up to 144 doses per year to maintain the target Hb levels of dialysis patients. In contrast, replacing Eprex with Aranesp, administered once a week, reduces the number of doses to 48 or less. This shift inevitably improves patient compliance and reduces the risk of medical errors associated with frequent drug administration. For medical staff, the extended dosing interval of Aranesp also alleviates the workload related to drug preparation and administration, minimizes the use of medical equipment, reduces the need for multiple drug storage locations, and saves time. These time savings can enable healthcare providers to allocate more time to focus on other aspects of CKD management, including patient counseling and education and addressing other risk factors. This study confirmed the advantage of the extended dosing interval of the long-acting Aranesp. It is consistent with findings from numerous articles published internationally in recent years, regardless of their specific efficacy results.^12–20^

It was found in this study that both Aranesp and Eprex exhibited similar incidences and compositions of adverse events. Notably, elevated blood pressure, hypertension, and pain at the injection site were the most frequently reported adverse events in both treatment groups (*P* > 0.05). Underlying CKD, comorbidities, and patients’ other treatments could be primary contributors to most of the reported adverse events. These results aligned with many other studies^12,19,20,33,34^ that reported similar adverse event profiles when comparing different ESA formulations. However, Bernieh et al. reported a significant increase in vascular access thrombosis in the Eprex group compared to the Aranesp group. However, the cause of this discrepancy remains unclear, as stated in their findings.^16^

In previous studies, the main advantages consistently reported for Aranesp were dosage reduction and cost savings.^13,15,32,35,36^ These findings are in line with the results of this study. We conducted a comprehensive analysis encompassing total costs, including drug expenses, iron costs, hemodialysis sessions, adjuvant therapy, monthly follow-up expenses, and costs associated with adverse events for both studied drugs. This analysis examined the costs and health outcomes of the two treatment regimens. Our cost-effectiveness analysis revealed that Aranesp was considered a dominant (cost-saving) strategy. However, in contrast to our findings, Bernieh et al. reported that the cost of Aranesp was double that of Eprex. They noted that their cost analysis took into consideration several factors, primarily the time-saving aspect related to the extended dosing interval, shifting from three times weekly to once weekly. They estimated that such a change could potentially save up to 350 hours of physician/nurse time per year in a hemodialysis center with 50 dialysis patients. These time savings could then be redirected to allow healthcare providers to devote more attention to various aspects of CKD management.^16^

## Strengths and Limitations

This study has several points of strength: the head-to-head comparison between the two most renowned ESA brands in Egypt and the prospective collection of the study data. The study assessed efficacy using various statistical methods, providing a comprehensive analysis. Additionally, the high adherence of all participating patients, as confirmed by the ESRD-AQ questionnaire, contributed to the robustness of the clinical response assessments. Including a cost-effectiveness analysis added depth to evaluating the best management regimen for anemia in CKD, considering various angles. On the other hand, the limitation of the study is that the relatively short study duration limited our ability to assess long-term safety issues associated with the treatments under investigation.

## Conclusion and Recommendations

In conclusion, our study demonstrates that Aranesp, with its extended dosing interval, is a more effective option than Eprex for achieving target Hb levels in Egyptian hemodialysis patients. This improved efficacy comes with comparable adverse effect profiles, significant cost-saving benefits, and a reduced workload for medical staff due to the extended dosing interval. Based on these findings, we recommend conducting further multicenter studies to validate our results and potentially implement them as guidelines for managing anemia in Egyptian hemodialysis patients.

## Ethics Approval

The study protocol was approved by the hemodialysis unit at The Memorial Souad Kafafi University Hospital, Giza, Egypt, and the ethical committee at the Faculty of Pharmacy, Helwan University, Cairo, Egypt (ethical committee approval number: 05H2022). The Clinicaltrial.gov registration ID is NCT05699109.

## Informed Consent

Each participant in the study outlined in the publication provided their informed permission.

## Consent for Publication

Not applicable.

## Conflict of Interest

The authors declare no competing interests.

## Author Contributions

Salma, Abdelhameed, and Hazem designed and prepared the study concept; all authors collected data. Salma and Amira analyzed and interpreted the data and drafted the article. All authors critically revised the article. All authors approved the article. Salma prepared statistics.

## Data Availability

The data supporting this study’s findings are available on request from the corresponding author. The data are not publicly available due to their containing information that could compromise the privacy of research participants.

## Acknowledgments

The authors would like to appreciate The Memorial Souad Kafafi University Hospital, Giza, Egypt, for the data arrangement and Dr. Sameh Elghandor, Consultant Nephrology at Alsheikh Zayed Specialized Hospital, for the great effort and help. The authors gratefully acknowledge cooperation of the patients who participated in this study.

## Figures and Tables

**Table 1.** Population data of both treatment groups.

	**Aranesp group**	**Eprex group**	**Total**	***P*-value**
Study population, n (%)	67 (52.8%)	60 (47.2%)	127	—
Gender, n (%)				
Male	47 (70.1%)	36 (60.0%)	83 (65.4%)	0.230*
Female	20 (29.9%)	24 (40.0%)	44 (34.6%)	
Age (years) (mean ± SD)	59.5 ± 10.2	62.4 ± 8.5	60.9 ± 9.5	0.099**
Weight (kg) (mean ± SD)	84.4 ± 11.5	84.7 ± 13.8		0.927**
Smoking status, n (%)				
Smoker(s)	9 (13.4%)	4 (6.7%)	13 (10.2%)	0.209*
Etiology, n (%)				
Hypertension	27 (40.3%)	30 (50%)	57 (44.9%)	0.655*
Diabetes	10 (14.9%)	9 (15%)	19 (15%)	
Hypertension and diabetes	20 (29.9%)	11 (18.3%)	31(24.4%)	
SLE	4 (6.0%)	4 (6.7%)	10 (7.9%)	
Unknown	6 (9.0%)	6 (10%)	10 (7.9%)	
Hepatitis status, n (%)				
HCV +ve	20 (29.9%)	18 (30%)	38 (29.9%)	0.985*
Vascular access, n (%)				
AVF	15 (82.1%)	54 (90.0%)	109 (85.8%)	0.202*
Permcath catheter	12 (17.9%)	6 (10%)	18 (14.2%)	

SLE, systemic lupus erythematosus; HCV, hepatitis C virus; AVF, arteriovenous fistula.

* *P*-values were obtained by a chi-square test.

** *P*-values were obtained by the Mann-Whitney U test.

**Table 2.** Biological parameters at baseline and for a 6-month study period in both treatment groups.

**Laboratory**	**Baseline**			**Mean value during the study period**		***P*-value**
	**Aranesp group *n* = 67**	**Eprex group *n* = 60**	***P*-value**	**Aranesp group *n* = 67**	**Eprex group *n* = 60**	
Hb [median (IQR)]	9.9 (9.3–10.3)	9.5 (9.2–10.3)	0.156**	10.8 (10.6–11.1)	10.3 (9.4–10.8)	< 0.001**
Creatinine (mean ± SD)	8.6 ± 2.0	8.1 ± 1.7	0.126*	8.4 ± 1.6	8.3 ± 1.4	0.525*
Ferritin [median (IQR)]	664.3 (284.4–944.5)	430.5 (184.5–1459.8)	0.377**	670.1 (354.1–940.0)	378.5 (200.2–1060.1)	0.241**
Iron [median (IQR)]	60 (49.0–69.0)	70.5 (47.3–82.5)	0.076**	61.0 (53.0–73.0)	68.8 (57.0–80.8)	0.079**
TIBC [median (IQR)]	222 (200.0–242.0)	223.5 (201.5–253.0)	0.646**	214.5 (196.0–233.0)	230.0 (202.1–267.1)	0.091**
TS (mean ± SD)	27.6 ± 12.2	31.7 ± 14.3	0.082*	28.8 ± 8.8	31.5 ± 12.1	0.161*
Calcium [median (IQR)]	8.8 (8.5–9.1)	8.8 (8.5–9.6)	0.345**	8.7 (8.4–9.0)	8.8 (8.5–9.2)	0.145**
Phosphate (mean ± SD)	4.9 ± 1.1	4.6 ± 0.9	0.178*	4.7 ± 1.0	4.4 ± 0.7	0.051*
Sodium [median (IQR)]	138.0 (137.0–139.0)	137 (136.2–139.0)	0.789**	138.0 (137.3–138.7)	137.9 (137.2–139.3)	0.442**
Potassium [median (IQR)]	4.9 (4.7–5.0)	4.9 (4.6–5.2)	0.683**	4.9 (4.7–5.0)	4.9 (4.8–5.3)	0.069**
iPTH [median (IQR)]	506.0 (322.0–598.6)	441.0 (301.8–603.0)	0.580**	469.5 (328.2–615.2)	428.5 (287.07–610.38)	0.454**
Albumin [median (IQR)]	3.9 (3.7–4.1)	3.8 (3.6–4.0)	0.123**	3.9 (3.7–4.1)	3.8 (3.7–3.9)	0.338**

IQR, interquartile range; TIBC, total iron binding capacity; TS, transferrin saturation; iPTH, intact parathyroid hormone.

* *P*-values were obtained by the independent t-test.

** *P*-values were obtained by the Mann-Whitney U test.

**Table 3.** Comparison between the two drugs at the baseline and the end of the study.

	**Mean rank**	***P*-value** *
Hb at baseline (first month)		
Aranesp, *N* = 67	68.4	0.156
Eprex, *N* = 60	59.1	
Hb at the end of the study period (sixth month)		
Aranesp, *N* = 67	80.6	< 0.001
Eprex, *N* = 60	45.4	

* *P*-values were obtained by the Mann-Whitney U test.

**Table 4.** Target Hb in each group.

**Total Hb measures in 6 months**	**Aranesp**		**Eprex**		***P*-value** *
Hb level (n, %)					
Low (< 10 g/dl)	70	17.4%	138	38.3%	< 0.001
Target (10–11.5 g/dl)	255	63.4%	182	50.6%	
High (> 11.5 g/dl)	77	19.2%	40	11.1%	
Total (762 measures)	402	100%	360	100%	

* *P*-values were obtained by a chi-square test.

**Table 5.** Adverse events in both treatment groups for a 6-month study period.

**Adverse event**	**Aranesp**	**Eprex**	***P*-value** *
Vascular access thrombosis	0 (0%)	3 (5%)	0.064
High blood pressure	6 (9%)	7 (11.7%)	0.615
Stroke	0 (0%)	1 (1.7%)	0.289
Myocardial infarction	1 (1.5%)	1 (1.7%)	0.937
Vomiting	2 (3%)	3 (5%)	0.560
Nausea	4 (6%)	5 (8.3)	0.604
Diarrhea	1 (1.5%)	0 (0%)	0.342
Edema	2 (3%)	1 (1.7%)	0.625
Pain at the site of the injection	7 (10.4)	9 (15%)	0.440

* *P*-values were obtained by a chi-square test.
